# NLRP3 inflammasome: a key player in the pathogenesis of life-style disorders

**DOI:** 10.1038/s12276-024-01261-8

**Published:** 2024-07-01

**Authors:** Rajath Ramachandran, Abdul Manan, Jei Kim, Sangdun Choi

**Affiliations:** 1 Department of Molecular Science and Technology, Ajou University, Suwon, 16499 Korea; 2 S&K Therapeutics, Ajou University Campus Plaza 418, 199 Worldcup-ro, Yeongtong-gu, Suwon, 16502 Korea

**Keywords:** Cell death and immune response, Metabolic disorders

## Abstract

Proinflammatory cytokines and chemokines play a crucial role in regulating the inflammatory response, which is essential for the proper functioning of our immune system. When infections or threats to the body’s defense mechanisms are detected, the innate immune system takes the lead. However, an excessive inflammatory response can lead to the production of high concentrations of cytotoxic molecules, resulting in tissue damage. Inflammasomes are significant contributors to innate immunity, and one of the most extensively studied inflammasome complexes is NOD-like receptor 3 (NLRP3). NLRP3 has a wide range of recognition mechanisms that streamline immune activation and eliminate pathogens. These cytosolic multiprotein complexes are composed of effector, adaptor, and sensor proteins, which are crucial for identifying intracellular bacterial breakdown products and initiating an innate immune cascade. To understand the diverse behavior of NLRP3 activation and its significance in the development of lifestyle-related diseases, one must delve into the study of the immune response and apoptosis mediated by the release of proinflammatory cytokines. In this review, we briefly explore the immune response in the context of lifestyle associated disorders such as obesity, hyperlipidemia, diabetes, chronic respiratory disease, oral disease, and cardiovascular disease.

## Study Reveals NLRP3 Inflammasome’s Impact on Lifestyle Diseases

NOD-like receptors (NLRs - proteins that help our immune system fight off harmful invaders) are vital for our health. Their function in T and B cells (types of white blood cells) is less clear. Scientists have found 22 kinds of NLRs in humans, which start different immune and inflammation responses. This study is a detailed review of NLRs, examining their structure, how they are activated, and their role in diseases like obesity, diabetes, and heart problems. It emphasizes that NLRs, particularly the NLRP3 inflammasome (a protein complex involved in inflammation), are key in lifestyle diseases by causing inflammation. The review proposes that focusing on NLRP3 could lead to new treatments for these diseases. This research is a big step in understanding how our natural immune system contributes to chronic diseases and offers potential for new treatments. Future research could further explore the complexities of NLRs and their potential as treatment targets.

This summary was initially drafted using artificial intelligence, then revised and fact-checked by the author.

## Introduction

**Fig. 1:**
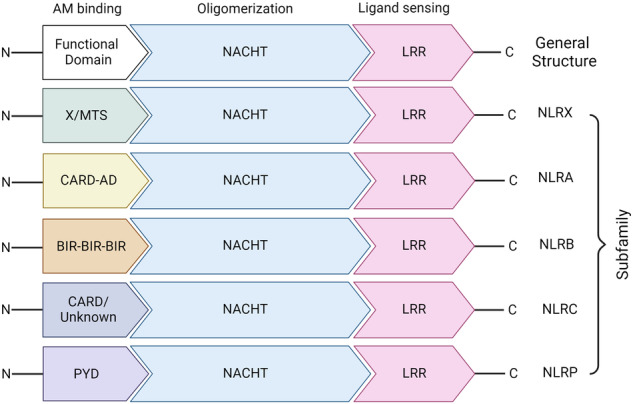
Structural insights into NLRs. NLRs consist of 5 subfamilies: NLRX, NLRA, NLRB, NLRC and NLRP. The ‘N’ represents the N-terminus, and ‘C’ represents the C-terminus. AM adaptor molecule, NACHT nucleoside triphosphatase (NTPase) domain, also known as nucleotide-binding and oligomerization domain, LRR leucine-rich repeat domain, X/MTS uncharacterized domain/mitochondrial targeting sequence, CARD-AD caspase recruitment domain-activation domain, BIR baculovirus IAP-repeat domain, PYD pyrin domain.

NOD-like receptors (NLRs) are receptors that bind to foreign molecules that may disturb the immune system. NLRs are mainly found in immune cells, such as neutrophils, dendritic cells and macrophages, but are less characterized in T cells and B cells. A total of 22 NLRs have been identified in humans. These receptors initiate various signaling pathways involved in immunity and inflammation. These pathways include mitogen-associated protein kinase (MAPK) signaling, nuclear factor-κB (NF-κB) signaling and inflammasome activation. Five subfamilies, namely, the NLRA, NLRB (NAIP), NLRC, NLRP and NLRX subfamilies, constitute the NLR family^1^. Generally, NLRs are composed of three domains: a C-terminal leucine-rich repeat (LRR) domain, a central nucleotide-binding oligomerization domain (NACHT) and a variable N-terminal effector domain (Fig. 1). Each domain has a unique function. LRR domains selectively recognize ligands, such as the stimuli that regulate invading pathogens. The NACHT domain functions in the oligomerization and activation of NLRs, and the heterogeneous N-terminus binds to the adaptor molecules and secondary messenger molecules to initiate the signaling pathway. NLRPs, except for NLRP10, share similar structural domains, especially the NACHT and LRR domains. The presence of the CARD and PYD protein-interacting domains is common in most NLRs. Nucleotide-binding oligomerization domain, LRR and pyrin domain-containing proteins (NLRPs) include several members: NLRP1 to NLRP9 and NLRPs from 11 to 14. NLRP1 is equipped with one extra CARD domain. NLRP10 is devoid of LRR, and thus, it contains only the NACHT and PYD domains. NLRCs contain a CARD domain, and NLRPs contain a PYD domain. However, NLRB does not contain either of these domains; rather, it contains the BIR domain. The mitochondrial targeting sequence and other domains, such as NLRC3, NLRX1 and NLRC5, are in the same category as the NACHT and LRR domains^2^. NLRA, which contains the N-terminal acidic domain, regulates antigen presentation (major histocompatibility complex class II). NLRB, which contains the baculoviral inhibition of apoptosis protein repeat (BIR)-like domain, is involved in host defense and determines whether cells die or survive. NLRs possessing a caspase activation and recruitment domain (CARD) at the N-terminus, called NLRC, interact with molecules that have similar structural domains. The N-terminal pyrin domain (PYD) of NLRP assembles and activates the inflammasome.

**Fig. 2:**
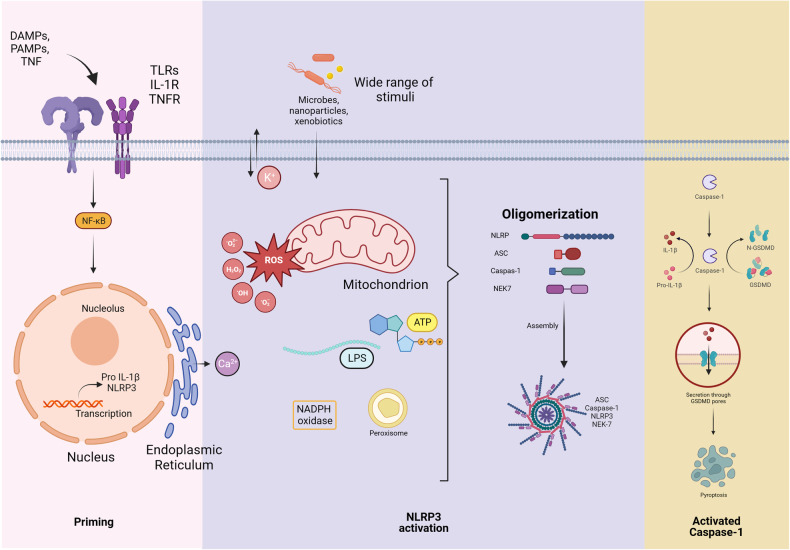
Overview of NLRP3 priming and activation. Cytokines and death signals through receptors such as TLRs and TNFRs mediate the transcription of NLRP3 proteins. Various factors, including xenobiotics, microbes, and ATP, increase the activity of the ROS-producing enzymes NADPH oxidase and peroxisomes. These events set off a cascade of reactions involving the generation of reactive oxygen species and alterations in calcium and potassium ion concentrations across the cell membrane that lead to NLRP3 priming and subsequent activation. In the next phase of the reaction, the assembled inflammasome activates caspase-1, resulting in the processing of pro-IL-1β and pro-IL-18. Finally, caspase-1 cleaves gasdermin D (GSDMD) and forms N-GSDMD, which attaches to the cell membrane, forming pores that facilitate the release of proinflammatory cytokines and induce pyroptosis. NLRP3 NOD-, LRR- and pyrin domain-containing protein 3, TLR Toll-like receptor, TNFR tumor necrosis factor receptor, ATP adenosine triphosphate, ROS reactive oxygen species, NADPH nicotinamide adenine dinucleotide phosphate, ASC apoptosis-associated speck-like protein containing a CARD, NEK7 NIMA-related kinase, IL-1β interleukin 1 β, IL-18 interleukin-18, GSDMD gasdermin D, N-GSDMD N-terminal gasdermin D.

The key determinants for the induction of the NLRP3 complex are pathogen-associated molecular patterns, danger-associated molecular patterns, reactive oxygen species (ROS), ion fluxes, lysosomal destabilization, ATP, and peptide aggregates^3^. Apoptosis-associated speck-like protein containing a CARD (ASC) is an adaptor protein that is recruited by NLRP3 once its leucine-rich repeat ligand-sensing region domain is activated. The oligomerization of recruited ASCs results in the formation of ASC specks. This is followed by the transfer of pro-caspase-1 to its CARD domain, which causes caspase-1 activation^4^. Following activation, this complex releases inflammatory cytokines, eventually leading to pyroptosis. Two sequential events, priming and activation, are necessary for the canonical activation of the NLRP3 inflammasome. The activation and nuclear translocation of NF-κB by pathogen-associated molecular patterns, such as the lipopolysaccharide priming signal, via Toll-like receptors leads to the transcription of inflammatory cytokine genes (Fig. 2). Procaspase-1 cleavage eventually results from the engagement of NLRP3 due to different factors^3^. Two enzymatically active caspase-1 subunits, p10 and p20, are produced as a result of the cleavage of procaspase-1. Then, interleukin-1, interleukin-18, and gasdermin D are converted to their mature forms by active caspase-1. On the cell membrane, mature gasdermin D creates pores that cause pyroptotic cell death and cytokine release. Interleukin-1 and interleukin-18 promote immune cell infiltration and increase NLR-mediated inflammation^5^. The cytokines produced mitigate infections or injured cells when inflammasomes are activated in response to a threat signal^6^.

**Fig. 3:**
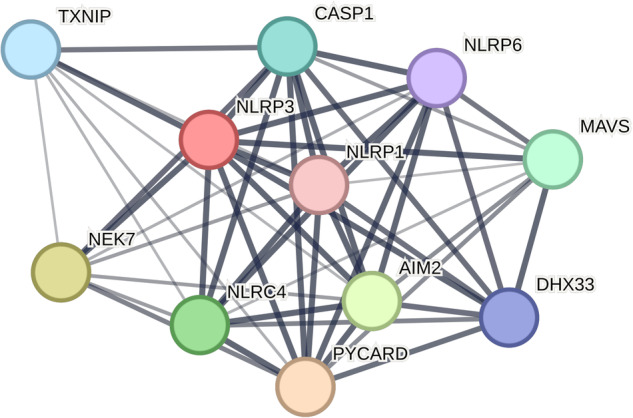
NLRP3 protein interaction network. NLRP3 protein interactions with other proteins are presented here. Network line thickness indicates the intensity of interaction. TXNIP thioredoxin-interacting protein, CASP1 caspase-1, NLRP3 NOD-, LRR- and pyrin domain-containing protein 3, NLRP6, NOD-, LRR- and pyrin domain-containing protein 6, NLRP1 NOD-, LRR- and pyrin domain-containing protein 1, NEK7 NIMA-related kinase, NLRC4 NLR family CARD domain containing 4, AIM2 absent in melanoma 2, PYCARD, PYD and CARD domain containing, DHX33 DEAH-box helicase 33, MAVS mitochondrial antiviral-signaling protein. The interaction network was generated using the STRING database.

At the genetic level, NLRP3 activation is regulated through posttranscriptional, posttranslational, and negative regulatory mechanisms^6^. The deubiquitinating enzyme BRCA1/BRCA2-containing complex subunit 3 triggers NLRP3 activation at the posttranslational level^7^. Upon ubiquitination of NLRP3, F-box L2^8^, TRIM31^9^, and MARCH7^10^ reduce NLRP3 inflammasome activation. Jun N-terminal kinase^11^ and protein kinase D^12^ increase NLRP3 activation via phosphorylation. Moreover, protein tyrosine phosphatase nonreceptor 22^13^ and protein phosphatase 2A^14^ are activated by dephosphorylation. Intracellular redox homeostasis is maintained by antioxidant protein thioredoxins. However, elevated intracellular ROS levels can induce the dissociation of TXNIP from thioredoxins and promote its binding to NLRP3, thereby activating NLRP3 (Fig. 3). ER stress is another factor that triggers NLRP3 activation. TXNIP overexpression^15^ and Ca^2+^ release into the cytosol due to endoplasmic reticulum stress and the unfolded protein response can harm mitochondria and aggravate ROS^16^. Other NLRP3 stimulators, such as cardiolipin and oxidized mtDNA, can also be generated in the cytosol by impaired mitochondria^17^. The lysosome is another organelle involved in NLRP3 activity. When ruptured, it releases cathepsin B into the cytosol, which activates NLRP3^18^. Numerous miRNAs (miR-223, miR-7, and miR-1929-3p) control NLRP3 inflammasome activity at the posttranscriptional level and sometimes inhibit NLRP3^19^.

Furthermore, extensive research suggests that the activation of NLRP3 is also regulated posttranscriptionally by lncRNAs, including nuclear enriched abundant transcript 1 (Neat1)^19^, MIAT^20^, Gm15441^21^, and Platr4^22^. Autophagy is the process by which damaged cells are eliminated, and NLRP3 is negatively regulated by autophagy. To inhibit and promote negative regulation, specific molecules can bind to elements of the NLRP3 inflammasome complex or molecules with similar functions. B-cell adapters for phosphoinositide 3-kinase^23^, PYRIN domain-only proteins 1^24^ and 2^25^, TRIM30^26^, heat shock protein 70^27^, and NLR family CARD-containing 3 protein^28^ are examples of established negative regulators of NLRP3.

## Association between NLRP3 and lifestyle diseases

**Fig. 4:**
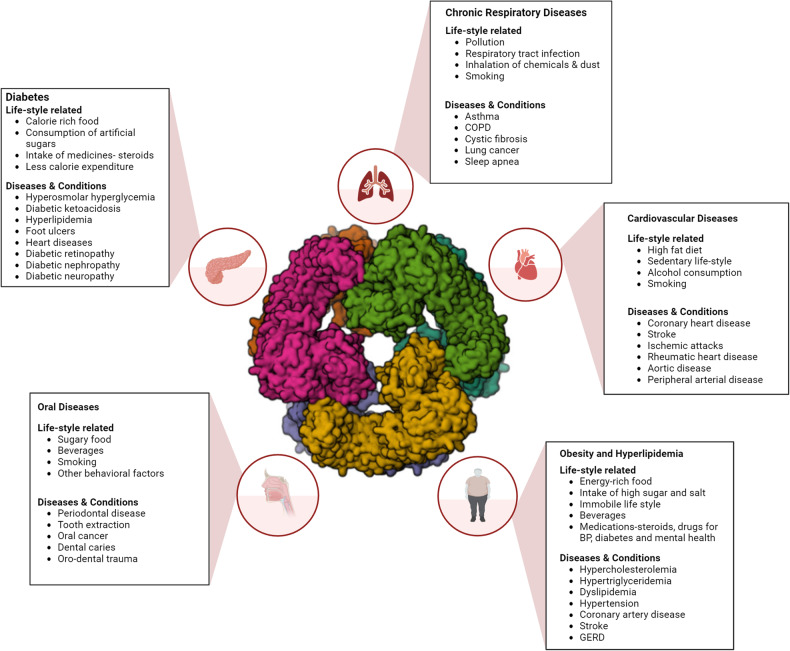
Association of NLRP3 with lifestyle disorders. The diagram depicts the life style habits those are linked to various disease conditions via NLRP3.

The NLRP3 inflammasome is an important sensor and indicator not only of inflammatory immune disorders but also of metabolic disorders, cardiovascular diseases, and respiratory ailments, which primarily constitute lifestyle diseases (Fig. 4). Lifestyle factors such as diet, exercise, and exposure to environmental pollutants are linked to the dysregulation of NLRP3, which contributes to the pathogenesis of these disorders. Understanding the molecular mechanisms underlying NLRP3 activation and its downstream effects is essential for the development of targeted therapeutic interventions. The key therapeutic implications of NLRP3 include its role in regulating cell death and proliferation. NLRP3 inflammasome assembly is closely intertwined with cell-mediated processes such as apoptosis, pyroptosis, and necroptosis. By modulating these pathways, inflammation can be mitigated, and tissue repair and regeneration can be promoted. This approach holds promise for preventing extensive damage associated with aging, disease pathologies, and other external factors. Therefore, the NLRP3 inflammasome is a promising therapeutic target for addressing the spectrum of lifestyle disorders that become prevalent with advancing age^29,30^. Researchers and clinicians are positioned to achieve substantial advancements in the management and prevention of diseases through the manipulation of cell death and regeneration processes intricately linked to NLRP3 activation. The ongoing, completed, withdrawn clinical studies related to the use of NLRP3 as a marker and a major player in lifestyle-associated noncommunicable diseases are listed in Table 1. Consequently, drug therapies targeting NLRP3 hold immense potential for enhancing overall health and extending life expectancy^31^.

**Table 1:** List of clinical studies on the direct and indirect roles of NLRP3 in lifestyle disorders.

Disorder	NCT no.	Study Title	Study Start	Study status
Obesity	NCT05482178	The Association of Resistance Exercise With the Inflammasome Activation in Obesity Subjects	2022	Recruiting
	NCT04275674	Inflammasome Activation and Cortisol Metabolism in Obese Women With Recurrent Miscarriage	2017	Recruiting
	NCT04315376	Exercise and Diet Intervention Attenuated Inflammation Through ASC Gene and Inflammatory Markers in Obese Adults (INDIEX)	2018	Completed
	NCT04814147	Role NLRP3 Inflammasome in Weight Loss Following Sleeve Gastrectomy in Morbidly Obese Patients (BARIAMITRI)	2021	Recruiting
	NCT05482178	The Association of Resistance Exercise With the Inflammasome Activation in Obesity Subjects	2022	Recruiting
	NCT04275674	Inflammasome Activation and Cortisol Metabolism in Obese Women With Recurrent Miscarriage	2017	Recruiting
	NCT04315376	Exercise and Diet Intervention Attenuated Inflammation Through ASC Gene and Inflammatory Markers in Obese Adults (INDIEX)	2018	Completed
	NCT01619215	The Effects of Bariatric Surgeries on Non-Alcoholic Fatty Liver Disease	2012	Unknown
Diabetes	NCT04432337	Role of Type 2 Diabetes in Potentiating the Inflammatory Response Post Extracorporeal Circulation After Cardiac Surgery (DT2CEC)	2021	Withdrawn
	NCT04485871	Targeting Risk Factors for Diabetes in Subjects With Normal Blood Cholesterol Using Omega-3 Fatty Acids	2019	Recruiting
	NCT06047262	Dapansutrile in Diabetes and Diabetes-Related Complications - Dapan-Dia (Dapan-Dia)	2023 (Estimated)	Not yet recruiting
	NCT04496154	Omega-3 to Reduce Diabetes Risk in Subjects With High Number of Particles That Carry “Bad Cholesterol” in the Blood	2013	Completed
	NCT02812238	Study to Evaluate the Effect of Nicotinamide Riboside on Immunity	2016	Completed
	NCT04510493	Canakinumab in Patients With COVID-19 and Type 2 Diabetes (CanCovDia)	2020	Completed
	NCT02964572	Effect of Sodium Glucose Cotransporter 2 Inhibitor on Inflammatory Cytokine in Type 2 Diabetes	2016	Completed
Chronic Respiratory Diseases	NCT03467035	Role of NLRP3 Inflammasome and Hypoxia in the Severity of Osteoporosis in Patients With Bronchiectasis	2017	Unknown
	NCT02471300	Effect of Fasting on the Asthma Inflammasome	2015	Completed
	NCT03467035	Role of NLRP3 Inflammasome and Hypoxia in the Severity of Osteoporosis in Patients With Bronchiectasis	2018	Unknown
Oral Diseases	NCT06075680	Effect of Periodontal Treatment on Inflammasome Proteins in Periodontal Diseases	2022	Active, not recruiting
	NCT05061511	Expression of Inflammasomes in Peri-implantitis and Periodontitis	2021	Recruiting
	NCT04450810	Role of NLRP3 in Periodontitis and Diabetes	2014	Completed
	NCT04127487	Quantification of NLRP3 (rs4612666) and CARD8 Gene (rs2043211) in Generalized Chronic Periodontitis Subjects	2019	Unknown
Cardiovascular diseases (CVDs)	NCT04766814	Epigenetic Analysis of Regulation of the Inflammasome-activating NLRP3 Gene in Monocytes From Atrial Fibrillation Patients and Controls	2021	Active, not recruiting
	NCT04269057	Change of NLRP3 Inflammasome Expression Level, Symptoms, and Functional Status in HFpEF Patients Treated With ARNI	2020	Unknown
	NCT05734612	The Role of Colchicine in Reducing The Rate of Myocardial Reperfusion Injury	2022	Enrolling
	NCT02122575	Effect of Fasting on the NLRP3 Inflammasome	2014	Completed
	NCT05330013	The Effect of Inflammation in Heart Failure	2022	Withdrawn
	NCT05880355	Advanced Cardiovascular Imaging of the Systemic Effects of Inflammasome Activation	2024 (Estimated)	Not yet recruiting
	NCT03745729	Allopurinol in Acute Coronary Syndrome	2019	Completed
	NCT05855746	Colchicine Versus Placebo in Acute Myocarditis Patients (ARGO)	2023 (Estimated)	Not yet recruiting
	NCT04232852	Probiotics and Systemic Inflammation in Patients With Metabolic Syndrome and High Cardiovascular Risk	2019	Withdrawn
	NCT02760914	Adipose Tissue and Inflammation in Coronary Heart Disease (ATICH)	2016	Active, not recruiting
	NCT02812238	Study to Evaluate the Effect of Nicotinamide Riboside on Immunity	2016	Completed
	NCT01928095	RNA Cloning and Visualization in Human Atherosclerosis	2013	Completed
	NCT05981144	Exploration of Clonal Hematopoiesis of Indeterminate Potential in Nonischemic Heart Failure With Reduced Ejection Fraction	2023	Completed
	NCT05756452	Linking Cardiac Autonomic Dysfunction and InfLammation in Patients With Acute Coronary Syndromes (CADILACS)	2023	Recruiting
	NCT05900947	Pollutants in the Atherosclerotic Plaque and Cardiovascular Events (APAChE)	2023	Completed

## Obesity and hyperlipidemia

**Fig. 5:**
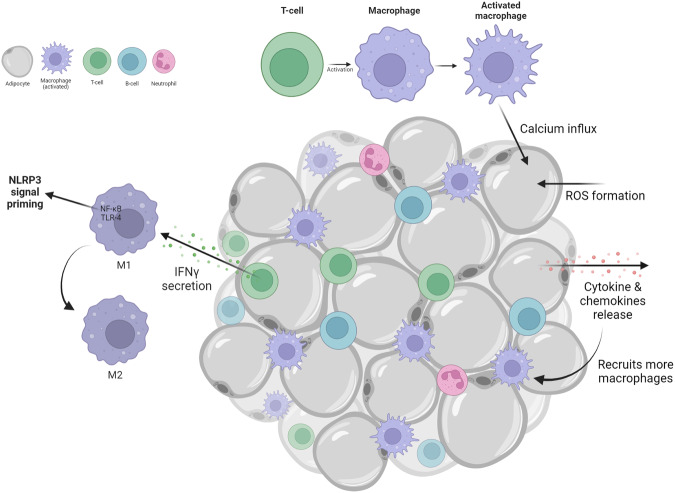
Significance of adipose tissues in NLRP3 priming. The conglomerates of fat droplets in between modified adipose tissue environment results in inflammation. The T cell response due to this event recruits more macrophages that eventually activates NLRP3 priming and IFN γ secretion in the downstream, and the simultaneous ROS formation and calcium influx promotes the release of proinflammatory cytokines.

The phenomenon of elevated lipid levels with clinically observable high cholesterol and triglyceride levels is known as hyperlipidemia. A total cholesterol level equal to or above 200 mg/dL, and a triglyceride level of 150 mg/dL or above is considered a high. Blood lipid levels must be controlled to prevent diabetes, stroke, heart disease, and other complications. Excessive body fat accumulation causes a chronic medical condition called obesity. A sedentary lifestyle and a high-fat diet can lead to further complications and weight gain. A person with a body mass index equal to or greater than 30 kg/m^2^ is considered obese. Chronic obesity impedes fat tissue function, and the concurrent high expression of proinflammatory cytokines is influenced by the induction of immune responses involving systemic inflammatory cytokines (Fig. 5). Obesity-induced metabolic disorders are characterized by adipose tissue inflammation and extracellular matrix remodeling^32^. A modified adipose tissue environment induces adipocyte hyperplasia and hypertrophy. This eventually leads to excessive breakdown of fat from adipocytes into fatty acids and the generation of ceramides that drive the efflux of cholesterol and other lipid forms, such as lipoproteins, from adipose tissue. These cholesterol crystals, ceramides, low-density lipoproteins, fatty acids, and fatty acid derivatives exacerbate the adverse conditions, leading to pyroptosis through the binding of macrophages to gasdermin D pores. In addition, long-chain fatty acid aggregates in nonadipose tissues cause lipotoxicity, which in turn leads to other effects, such as mitochondrial stress and inflammation^33^. The formation of cholesterol crystals and fatty acid derivatives also triggers TLR4 signaling via macrophages^34^. The activation of macrophages results in calcium influx into adipocytes, ROS formation, and the release of cytokines (TNF-α and IL-6) and chemokines (MCP-1)^35^. The chemokine MCP-1 recruits more macrophages, and interferon-gamma (IFN-γ) secreted from T cells polarizes macrophages toward the active proinflammatory (M1) state instead of the anti-inflammatory state (M2). The activation of NF-κB occurs as a consequence of these events, which intervene in NLRP3 signal priming (Fig. 5). Inflammasome priming, which induces the NLRP3 signaling pathway, is initiated by endogenous cellular components and cytokines, including lipopolysaccharide, and elevates pro-IL-18, pro-IL-1β, and NLRP3 protein levels^36^. Similarly, danger-associated molecular patterns and pathogen-associated molecular patterns produce inflammasome signals (Fig. 2). In obese individuals, lipid droplets are common in myocytes and hepatocytes. Intracellular fat droplets are stored in these lipid droplets and cause inflammation and metabolic disorders^33^. Accumulated fat causes macrophage infiltration, altering the tissue microenvironment and releasing inflammatory agents. Changes in the tissue microenvironment depend on several other factors, such as the intracellular ATP concentration, mitochondrial oxidation, the formation of ROS, and lysosomal destabilization^37^. Another factor that leads to the formation of mitochondrial ROS is impaired cellular respiration or other stress conditions, such as inflammation. This event is followed by the release of NLRP3, resulting in the propagation of inflammatory signals. Kursawe et al. reported that NLRP3 and other inflammasome cytokines were more abundant in obese adolescents than in healthy control individuals^38^. Vandanmagsar et al. reported that in obese patients with type 2 diabetes mellitus, weight loss resulted in reduced inflammation in subcutaneous adipose tissue and a simultaneous decrease NLRP3 inflammasome expression^39^. The relationship between elevated NLRP3 levels and obesity indicates that these cascade events occur as a result of extracellular matrix remodeling of visceral adipose tissues.

Because of these processes, an osmotic imbalance occurs as the concentration difference across the membrane allows proinflammatory chemical molecules to target the site. A matter of concern is the final result of cell death, which is either necrosis or pyroptosis. The excessive secretion of cytokines continues throughout this cycle and is considered a disease. Indications for metabolic diseases, such as visceral adiposity, glycemia, increased blood pressure, and high blood cholesterol levels, are associated with obesity-induced tissue inflammation, which is characteristic of type 2 diabetes and insulin resistance^40^. The mRNA expression levels of NLRP3 and the macrophage marker Cd11c in adipocytes showed that obesity is related to NLRP3 induction, which can cause a lipid-loaded macrophage response^39^. Thus, obesity promotes NLRP3 and macrophage-mediated T-cell activation in adipose tissues, which are also responsible for insulin sensitivity^41^. Visfatin is an important harmful adipokine in obesity and its associated diseases. It possesses proinflammatory features and mediates endothelial inflammation and injury through inflammasomes^42^. Additionally, circulating saturated free fatty acids are a danger-associated molecular pattern that is involved in TLR4-mediated NLRP3 inflammasome activation^43^, which can also cause insulin resistance. Studies on insulin resistance have demonstrated the ability of NLRP3 to recognize obesity-induced danger-associated molecular patterns^39,44^. Obese patients with chronic visceral adipose tissue inflammation exhibit increased NLRP3, NLRP1, and NLRP6 protein levels^45^. The liver detects increased levels of cytokines and secretes C-reactive proteins. C-reactive protein is a key biomarker of inflammation and has been observed in most metabolic disorders. Moreover, inflammation, which contributes to disease progression, is a major concern^33^.

C-reactive protein initiates NF-κB signaling, which induces proinflammatory cytokine secretion and thereby amplifies inflammatory conditions^46^. Several studies have clarified the relationship between obesity-related inflammation and NLRP3 and the involvement of this relationship in various disease states. Finucane et al. provided mice a high-fat diet containing monosaturated and saturated fatty acids and observed increased levels of the cytokines NLRP3, Casp-1, and IL-1β^47^. COVID-19 patients with obesity are more prone to metabolic disturbances, especially meta-inflammation (metabolic inflammation) than those without obesity. Obesity and diabetes are closely associated with meta-inflammation; NLRP3 is a key regulator of these inflammatory changes. In COVID-19 patients, the inflammatory immune response during the cytokine storm in the presence of NLRP3 activators worsens health conditions, such as multiple organ failure and internal bleeding, leading to mortality. NLRP3 activators are involved in various pathophysiological conditions of obesity, including hyperlipidemia^48^, meta-inflammation, and surges in the levels of inflammatory cytokines, adipokines, and other chemokines. The clinical records of COVID-19 patients from different parts of the world are important data sources that highlight the need for the intensive care for severe pneumonia caused by COVID-19-associated cytokine release syndrome in young obese patients^49,50^.

## Diabetes and insulin sensitivity

Diabetes is a lifestyle disease caused by impaired glucose metabolism and insulin secretion. The fatality rate of patients with diabetes is noteworthy. In 2019, approximately 1.5 million patients died worldwide^51^, and by 2045, the global number of patients with diabetes is expected to increase to 783 million. Obesity and obesity-associated type 2 diabetes are considered metabolic syndromes because of their chronic inflammatory nature involving NLRP3 inflammasome activation^52^. T cells in adipose tissue are regulated by the NLRP3 inflammasome under obese conditions. Inflamed adipocytes attract T-cell populations via chemotaxis, which is believed to cause tissue inflammation and insulin resistance^53^. Insulin resistance is defined as the inability of insulin to stimulate biological activity, resulting in impaired glucose metabolism. A similar phenomenon that occurs in neurons results in brain insulin resistance. This may be due to unresponsiveness to insulin or inadequate brain insulin action. Obesity and type 2 diabetes affect peripheral insulin signaling and trigger inflammation. Physiological changes such as increased oxidative stress, impaired lipid metabolism, hypoxia, and finally mitochondrial dysfunction are some of the conditions that result in elevated levels of chemokines, proinflammatory adipokines, and cytokines^54^. Among cytokines, TNF-α, IL-6, IL-18, and IL-1β are the most important in insulin resistance conditions. Excessive IL-1β production results in insulin resistance because of a decrease in insulin receptor substrate-1 phosphorylation and reduced *IRS-1* expression^55^. These findings indicate a correlation with type 2 diabetes mellitus. Inhibition of this signaling pathway has also been shown to result in good glycemic control and reduced proinflammatory cytokine levels^56^. An improvement in insulin sensitivity was observed during the depletion of NLRP3 in adipose tissue, which in turn reduced the levels of markers of systemic inflammation^39^. Several studies in rodent models have demonstrated the role of NLRP3 in glucose metabolism. Wen et al. reported a positive effect of NLRP3 deficiency on blood glucose levels and insulin action in mice, even when provided a high-fat diet^44^. In mouse model studies, glucose intolerance and insulin insensitivity increased the levels of NLRP3, ASC, caspase-1, or IL-1β^57^. A significant increase in the gene expression of IL-18, IL-1β, and NLRP3 has been observed in patients with type 2 diabetes^45^. The molecular signals that cause insulin sensitivity depend on the NLRP3 inflammasome-dependent processing of IL-18 and IL-1β during posttranslational modifications that result in chronic inflammation^39^. In a comparison between NLRP3 knockout mice and normal chow-fed control mice, the danger signals from IL-18 and IFN-γ were promoted by the activation of NLRP3 in obese conditions, and their consistent expression led to insulin resistance^39^.

## Cardiovascular diseases

Medical disorders affecting the heart and blood vasculature are generally referred to as cardiovascular diseases^58^. These include coronary heart disease, stroke, ischemic attack, rheumatic heart disease, aortic disease, and peripheral arterial disease (Fig. 4). Cardiovascular diseases account for 32% of global deaths, and 85% of these fatalities are due to strokes or heart attack^59^. Important risk factors include a high-fat diet, a lack of physical activity, and the consumption of alcohol and tobacco. The physical indicators include hypertension, increased blood glucose levels, high blood cholesterol and triglyceride levels and obesity. Krishnan et al. reported that systemic hypertension was associated with increased levels of IL-1β^60^. The inhibition of NF-κB via the downregulation of NLRP3 and caspase-1 indicated an antihypertensive effect that significantly reduced the amount of inflammatory cytokines^61^. The presence of IL-1β in the circulation is an essential indicator of NLRP3 inflammasome activation. Angiotensin II is a key regulator of blood pressure that is also closely associated with the release of proinflammatory agents and particularly activates the NLRP3 inflammasome^62^. Angiotensin II induces cardiac fibrosis, and the fibrotic process slows upon NLRP3 inhibition^62^. A cardio-protective outcome was achieved after reducing hypothalamic sympatho-excitation caused by the inhibition of IL-1β, which in turn altered blood pressure through a cascade of events such as attenuation of the renin-angiotensin system and decreased oxidative stress^63^. NLRP3 inflammasomes participate in acute/chronic inflammatory states followed by cell death, which is correlated with the severity of cardiovascular diseases and their risk factors^64^. Atherosclerotic plaques progress to the lesion stage through a number of phenomena, such as inflammation, oxidative stress, endoplasmic reticulum stress, impairment of mitochondria, and rupture of lysosomes^65^. This not only leads to the secretion of pro-IL-1β and other proinflammatory cytokines but also activates NLRP3 both directly and indirectly.

The bilateral association distinguishes between lipid-related and inflammation-related contributions to atherosclerotic diseases^66^. Cholesterol activates the NLRP3 inflammasome in atherosclerotic lesions, mostly through the production of IL-1, and plays a significant role in the development of cardiovascular disease^67^. The pathophysiology of heart failure involves the activation of inflammatory pathways as well as interactions with other pathways that control the function of myocytes, vasculature, and the extracellular matrix^68^. The purinergic macrophage receptor P2RX7 is highly expressed in atherosclerotic plaques, especially in foam cells, and it induces the accumulation of lipids, cholesterol crystals, and necrotic tissue in the extracellular sites of blood vessels. P2RX7 increases protein kinase R phosphorylation, thereby controlling NLRP3 complex formation and catalyzing atherogenesis by releasing chemoattractants and inflammatory signals^69^.

The calcification of blood vessels and atherosclerotic plaques is related to the deposition of calcium phosphate crystals, which activate the inflammasome process and initiate the caspase-1 pathway as well as the cleavage of IL-1^70^. Several studies have established a relationship between cholesterol crystals and NLRP3 expression. The sesquiterpene arglabin is known for its anti-NLRP3 activity, which inhibits the production of IL-1β, caspase-1, and IL-18 and induces autophagy in macrophages. Moreover, it reduces the size of atherosclerotic lesions and the levels of oxidized low-density lipoproteins and total cholesterol-triglycerides^71^. NF-E2-related 2 (Nrf2) influences the activation of NLRP3 in the presence of cholesterol crystals in the same manner as it does in atherosclerosis, and this finding has been verified by the quantification of IL-1β under conditions of Nrf2 expression and silencing^72^. Thus, the contribution of inflammatory cell infiltration and oxidative stress to the development of atherosclerosis has been established. Lipopolysaccharides, including lipoproteins, induce processes that increase the expression of plaque adhesion molecules, such as lectin-like oxidized low-density lipoprotein receptor-1. This receptor recruits leukocytes to the lesion, and liposaccharides promote ROS generation^73^. This event continues until mitochondrial DNA damage occurs, and then, cell autophagy and inflammasome activation occur^74^. Sandanger et al. observed intensified NLRP3 inflammasome signals in postmyocardial infarction myocardial fibroblasts, which influenced the infarct size and resulted in ischemic reperfusion^75^. Both the ASC and pro-caspase-1 of the NLRP3 inflammasome contain caspase activation and recruitment domain 3 (CARD3), which facilitates cardiac remodeling and ventricular dysfunction subsequent to myocardial infarction^76^. Moreover, the availability of additional validated data regarding the role of NLRP3 in myocardial remodeling further strengthens previous findings^77^.

The inflammasome-derived inflammatory cysteine protease caspase-1 degrades smooth muscle cell contractile proteins, affecting the biomechanical function of the aorta in aortic aneurysms. The outcome was validated using the inflammasome inhibitor glyburide, which alleviates the development of aortic aneurysm and dissection^78^. Abnormal NLRP3 induction results in the reconfiguration of myocytes and ultimately leads to improper function and the release of damaged mitochondrial DNA that binds to NLRP3. This triggers the NF-κB pathway associated with inflammasome activation^77^. Cardiac therapeutic agents such as nitroglycerides and nitrogen oxides, which release nitric oxide at local sites, cause venodilation^79^. The same nitric oxide has detrimental effects on NLRP3 inflammasome activation^80^. Bruton’s tyrosine kinase is a key mediator of B-cell receptor signaling. Bruton’s tyrosine kinase directly interacts with NLRP3 and ASC, initiating ASC oligomerization and caspase-1 activation^81,82^. Murthy et al. reported the presence of Bruton’s tyrosine kinase in platelets; thus, it promotes inflammasome activation, leading to thrombus formation^83^. Similarly, the platelet integrin αIIbβ3 was confirmed to be controlled by NLRP3 through interfering with platelet hemostasis and clot formation^84^. NLRP3 promotes fibrosis and profibrotic states in different ways, including through cAMP signaling and the Smad pathway^85^. One of the prevalent factors in heart failure is dilated cardiomyopathy (left ventricular dilatation with contractile dysfunction), which involves pyroptosis in cardiomyocytes via the integrated induction of NADPH oxidase (NOX1 and NOX4) and dynamin-related protein 1 (Drp1) via activation of the NLRP3 inflammasome^86^. Reperfusion injuries occur during ischemic heart disease, and their effects are intensified by inflammasomes. In a study conducted by Audia et al., the use of VX-765, a caspase-1 inhibitor, decreased the infarct size and preserved ventricular function, indicating its cardioprotective ability despite inflammasome activation^87^.

Butts et al. demonstrated the epigenetic control of ASC with reference to ASC methylation, mRNA, IL-18, and iNOS levels among physically active groups and patients with heart failure and concluded that the regulation of ASC could improve health status in the course of the disease^88^. CaMKIIδ participates in heart failure etiology and stimulates inflammasomes that culminate in myocardial fibrosis^89^. Septic cardiomyopathy, Chagas cardiomyopathy, idiopathic dilated cardiomyopathy, genetic structural cardiomyopathy, and autoimmune myocarditis are characterized by the activation of NLRP3 in cardiac fibroblasts^89^. In light of this research, NLRP3 inflammasome activation can be easily correlated with the risk of cardiovascular disease.

## Chronic respiratory diseases

Chronic respiratory diseases are a group of diseases that cause breathing issues, including problems in the lungs and airways, and they occur in various forms, including asthma, chronic obstructive pulmonary disease (COPD), cystic fibrosis, lung cancer, and sleep apnea^90^. Chronic respiratory diseases have several risk factors that contribute to their development and progression. The principal risk factors include exposure to tobacco smoke, ambient air pollution, inhalation of occupational chemicals and dust, and a history of recurrent lower respiratory tract infections during childhood (Fig. 4). Respiratory distress conditions, such as inflammation of the respiratory system due to injuries or respiratory diseases, have been implicated in NLRP3 inflammasome activation^91^. Exposure to asbestos, silica particles, and other particulate matter can also induce respiratory inflammatory responses via the NLRP3 inflammasome, leading to pulmonary fibrosis^15^. The levels of IL-1β in sputum and bronchoalveolar lavage fluid were higher in patients with asthma than in normal healthy adults, suggesting that the NLRP3 inflammasome may be involved in respiratory inflammation^92,93^. The association of NLRP3 inflammasome overexpression with restricted airflow to the respiratory tract, emphysema, and lung inflammation has been reported in studies on COPD^15,94^. Public data have indicated that persistent exposure to cigarette smoke, particulate matter, and other substances resulting from air pollution often causes airway passage inflammation and related diseases such as fibrosis and alveolar abnormalities in susceptible individuals^95^. This results in clinical manifestations such as a rigid chest, decreased lung capacity, dyspnea, cough, and purging of sputum. Yu et al. observed that patients with acute exacerbated COPD had high levels of inflammatory cytokines in their blood and sputum^96^. In summary, the release of inflammatory cytokines is related to immune mechanisms involving blood cells. In a study involving smokers in the recovery and stable stages of COPD, the white blood cell count was a notably decreased in patients at the acute exacerbation stage, although it was higher than that in smokers. Moreover, the levels of NLRP3, ASC, caspase-1, IL-1β, and IL-18 in peripheral blood cells and alveolar tissues were greater in all patients with COPD than in smokers. Further analysis of serum and bronchoalveolar lavage fluid showed significant increases in the levels of IL-18 and IL-1β in patients with COPD. Overall, the local and systemic airway passage inflammation caused by COPD can result in NLRP3 activation^97^. Cigarette smoke extract has been shown to increase IL-β, caspase-1, and NLRP3 protein levels in a concentration-dependent manner in advanced COPD models^98^. The NLRP3-specific siRNA (siNLRP3) reduced both NLRP3 and caspase-1 at the mRNA and protein levels. In addition, siNLRP3 also reduces IL-18 and IL-1β mRNA levels^99^. These studies suggest that NLRP3 activation exacerbates COPD.

Asthma is another chronic airway inflammatory disease with an etiology involving T cells and eosinophil inflammation, and CD4 + T-cell aggregation plays a role in this condition. Like other respiratory diseases, such as COPD, asthma is characterized by severe symptoms, including a rigid chest, cough, breathing difficulty, and wheezing, usually in the early morning or night. The formation of localized cytoplasmic Charcot–Leyden granules as a result of intense eosinophil degranulation or inflammatory responses leads to the assembly of the NLRP3 inflammasome and the concurrent release of IL-1β^100^. Other factors, such as follistatin-like protein 1, can act indirectly as modulators of the NLRP3 inflammasome in hyperresponsive airway conditions. When follistatin-like protein 1 is produced, it interacts with numerous signaling pathways that induce the expression of various cytokines and chemokines^101^. The airway mucins MUC5AC and MUC5B cause mucus plugging and obstruct air passage. Wang et al. (2021) studied the effect of Fstl1^±^ on OVA-induced asthmatic mice and macrophages and reported that Fstl1^±^ reduced the pathological progression of the disease by appreciably inhibiting OVA-induced MUC5AC and mucin secretion. Furthermore, the infiltration of inflammatory cytokines, including NLRP3 and IL-1β, into the BALF decreased with the inhibition of bronchial follistatin-like protein 1^102^. In summary, follistatin-like protein 1-induced NLRP3/IL-1β signaling initiates an inflammatory response, followed by the release of various cytokines that can provoke severe asthmatic symptoms. The airway allergen Der f1 from house dust mites is highly cross-reactive to asthmatic responses because of the NLRP3 inflammasome and IL-1β production in human bronchial epithelial cells^103^. Consistent with these findings, a clinical study by Wood et al. revealed that the expression of NLRP3 and IL-1β was upregulated in patients with asthma, suggesting its relevance in the design of anti-asthmatic drugs^104^.

## Oral diseases

Oral diseases include a number of oral pathologies and medical conditions, such as periodontal disease, tooth loss, oral cancer, dental caries, and orodental trauma. Oral health is associated with the prevalence of other chronic diseases, including diabetes and cardiovascular diseases^105^. Furthermore, oral diseases are significantly associated with adverse behavioral factors, such as tobacco use and the consumption of sugary foods and beverages (Fig. 4). Complexes of bacterial species that secrete inflammatory cytokines cause periodontal infections. Initially, as a defense response, these cytokines help eliminate pathogenic microbes. However, excessive levels of inflammatory cytokines are contraindicated owing to the destruction of collagen, resorption of the alveolar bone, and loss of periodontal attachment^106^. Chronic inflammatory conditions known as periodontitis affect the alveolar bone between the teeth and the surrounding connective tissue. These effects cause a gradual loss of the periodontium and the development of periodontal pockets and gingival recession. This cascade leads to tooth extraction. NLRP3 influences alveolar bone loss in ligature-induced periodontitis via osteoclast differentiation. MCC950, a specific NLRP3 inhibitor, suppresses alveolar bone loss by decreasing IL-1 activation and osteoclast differentiation in ligature-induced periodontitis^107^. This finding supports the involvement of NLRP3 in periodontitis. Oropharyngeal candidiasis and oral leukoplakia are two main oral medical conditions that are increasingly prevalent in the smoking population and are implicated in the generation of a number of oxidizing agents, including ROS, in the oral cavity. Smoking can alter the oral microbiome environment, harming the structural integrity and function of the epithelial barrier of the mouth, which acts as the body’s first line of protection against fungal infections^108^. Treatment of liposaccharide-G–induced inflammatory cellular models with the natural antioxidant ascorbic acid reduced the activity of TLR4, MyD88, NF-κB, NLRP3, caspase-1, and IL-1β^109^. This finding demonstrated the role of antioxidants in the immunomodulation of inflammatory pathways. Smoking results in oxidative stress and an imbalance in the cellular redox state, which in turn indirectly suppresses the phagocytic activity initiated by the immune system and impacts chemotaxis, kinesis, and cell signaling^110^. Consistent with the results of previous studies, Ye et al. demonstrated a connection between smoking and the suppression of *Candida albicans*-induced NLRP3 inflammasome activation and proinflammatory cytokine production via Nrf2 signaling^111^. In vitro studies revealed that Nrf2 not only inhibits the secretion of proinflammatory cytokines (IL-1, IL-6, TNF-α, and IL-18) but also negatively regulates the expression levels of the NLRP3 inflammasome and caspase-1 p20. These effects destabilize the antifungal defenses of the oral mucosa and induce oropharyngeal candidiasis and oral leukoplakia^111^. Thus, Nrf2 is an important target protein that regulates NLRP3 inflammasome activation.

## Other disorders

Multiple disorders, particularly Crohn’s disease^112^, rheumatoid arthritis^113^, systemic lupus erythematosus^114^, aspirin-induced asthma^115^, HIV infection^116^, celiac disease, and type 1 diabetes^117^, have been linked to *NLRP3* activity. The formation of the NLRP3 inflammasome is characteristic of enhanced NLRP3-NEK7 interactions, especially those interactions caused by genetic alterations in autoinflammatory diseases^118^, including chronic infantile neurological, cutaneous, and articular syndrome and cochlear autoinflammatory disease^119^. According to Zheng et al., *NLRP3* polymorphisms, particularly at the 3′UTR and in the G allele, cause enhanced mRNA stability, and increases in the frequency of these genotypes are associated with insulin resistance and type 2 diabetes^120^. The inflammatory etiology of nonalcoholic fatty liver disease indicates that the NLRP3 inflammasome is involved in disease progression^121^. In a study on diet-induced steatohepatitis^39^, Nlrp3-knockout mice were protected against the disease. However, mice with hepatosteatosis showed increased NLRP3 levels^122^, indicating that the NLRP3 inflammasome plays a role in liver disorders. Several SARS-CoV-2 proteins, including ORF3a, ORF8a, and E, increase the pathogenicity and cytokine storm in COVID-19^123,124^ by acting as NLRP3 inducers^125^. The unique SARS-CoV protein domain triggers the NLRP3 inflammasome, which causes a significant increase in IL-1β expression. Melatonin antagonizes NLRP3 inflammasome activity by restoring Nrf2 and SIRT1 protein levels, thereby restoring lupus lesions to their normal state^126^. In addition, the antioxidant melatonin demonstrated a suppressive effect on NLRP3 in both immune and nonimmune cells. This suppression prevented the formation of ROS and restored the expression of a microRNA that inhibits the expression of NLRP3 mRNA (miR-30e). This explains why melatonin-treated mice following radiation had low levels of NLRP3, ASC, and IL-1β^127^. Several studies have shown that the inflammasome worsens the damage from ischemic stroke^128,129^. Yang et al. reported NLRP3 overexpression in the postischemic brain^130^. The NLRP3 inflammasome and its components promote prothrombotic characteristics during disease progression, along with their consequent therapeutic implications^131^. The findings on neuroinflammation and intracranial injury associated with the use of inflammasome inhibitors or the blockade of other components in the pathway are consistent with those of previous studies that support the use of the NLRP3 inflammasome as a potential therapeutic target that needs to be considered in almost every inflammatory disease condition^132,133^.

## Clinical trial drug candidates for NLRP3

### Inhibitors

#### Dapansutrile (OLT1177) [CAS: 54863-37-5]

Dapansutrile is a selective NLRP3 inhibitor candidate in the clinical development stage that was developed by Olagtec Therapeutics New York. It has successfully completed a phase 1 clinical trial and was found to be safe in humans. Currently, the molecule has passed phase 2 clinical trials for acute gout flares (NCT01768975 and NCT02104050). Randomized double-blind phase 2 studies using dapansutrile topical gel for osteoarthritis pain have been conducted, and a phase 1b study of orally administered dapansutrile for heart failure has been completed (NCT03534297).

#### Selnoflast (RG6418) [CAS: 2260969-36-4]

RG6418, also known as selnoflast or Somalix (formerly IZD334), is a potent small molecule that inhibits the NLRP3 inflammasome^134^, and it has completed a phase 1 clinical study (NCT04086602). This compound was developed by Inflazome UK Ltd. and subsequently acquired by Roche. The chemical structure of this orally administered molecule is similar to that of MCC7840 (Emlenoflast) and MCC950, and its anti-inflammatory effect on chronic inflammatory diseases with NLRP3 activation has been demonstrated in pharmacological studies.

#### Emlenoflast (MCC7840, Inzomelid) [CAS: 1995067-59-8]

Emlenoflast (MCC7840, formerly IZD174) is a small molecule inhibitor of the NLRP3 inflammasome that has anti-inflammatory effects. In 2020, Roche acquired this orally bioavailable drug candidate capable of crossing the blood‒brain barrier, which was discovered by Inflazome UK Ltd. Phase 1 interventional studies on the safety, efficacy, PD and PK of this molecule were completed in cryopyrin-associated periodic syndrome patients in 2020 (NCT04015076).

#### NT0796 [CAS: 2272917-13-0]

NT0796, which was discovered by NodThera Ltd., is a prodrug of NDT19795 that selectively inhibits NLRP3-driven diseases. This clinical candidate is in a phase 1/phase 2 interventional study that started in 2023 and is estimated to be completed in 2024 for cardiovascular diseases (NCT06129409). This molecule has been shown to effectively penetrate the blood–brain barrier with good pharmacodynamic and pharmacokinetic profiles and is currently undergoing clinical evaluation for both peripheral inflammatory diseases and central nervous system inflammatory disorders.

#### VTX2735

VTX2735 is an orally administered small molecule inhibitor of NLRP3 designed by Ventyx Biosciences for systemic inflammation without any effect on the CNS. As of the current date, the structure has not been disclosed. In 2022, the inhibitor passed a phase 1 clinical trial followed by an ongoing phase 2 study for safety and clinical activity in patients with cryopyrin-associated periodic syndrome (CAPS), a condition caused by an NLRP3 gene mutation (NCT05812781).

#### VTX3232

VTX3232 is another novel oral selective inhibitor of NLRP3 developed by Ventyx Biosciences that can cross the blood‒brain barrier for the treatment of neurodegenerative diseases, including Parkinson’s disease, multiple sclerosis, Alzheimer’s disease and amyotrophic lateral sclerosis. The company announced the initiation of dosing for phase 1 trials in 2023, and the results have yet to be reported in 2024.

#### ZYIL1 [CAS: 2455519-86-3]

ZYIL1, which was designed by Zydus Lifesciences Ltd., selectively targets NLRP3 and hinders NLRP3-induced ASC oligomerization, thereby inhibiting it. Phase 1 interventional studies via oral capsules were completed in 2022 to ensure the safety, tolerability, pharmacokinetics, and pharmacodynamics of the drug candidates (NCT04731324, NCT04972188). The company has conducted a phase 2a prospective open-label study for cryopyrin-associated periodic syndromes (CAPS) that was completed in 2022 (NCT05186051). Another placebo-controlled, randomized, multicenter, double-blind phase 2 study for the treatment of amyotrophic lateral sclerosis (ALS) is ongoing and is expected to be completed on 2024 (NCT05981040).

#### MCC950 (CRID3) [CAS: 210826-40-7]

MCC950 (CRID3 or CP-456,773) is a withdrawn small molecule chemical produced by Pfizer that was initially used as an interleukin-1 inhibitor. Subsequently, it was found to directly bind to the NACHT domain of NLRP3 and inhibit its ATPase activity. It does not block NLRC4, NLRP3 priming, AIM2 or TLR signaling. In vivo studies have indicated its application for multiple NLRP3-driven diseases, such as type 2 diabetes, atherosclerosis, CNS disorders such as Alzheimer’s and Parkinson’s disease, inflammation and cancer^135^. After phase 1, the molecule advanced to phase 2 trials for rheumatoid arthritis with a relatively higher dose of 1200 mg per day, but the study has been limited due to drug-induced hepatic toxicity^136^.

#### Agonist

##### BMS-986299 [CAS: 2242952-69-6]

BMS-986299 is an NLRP3 agonist developed by Bristol-Myers Squibb for advanced solid tumors with an EC50 of 1.28 μM. This molecule activates NLRP3 and induces IL-8 formation to recruit natural killer cells to solid tumor cells. A phase 1 interventional study of this therapeutic candidate started in 2018 (NCT03444753) but terminated early due to the COVID-19 pandemic^137^.

#### Antagonist

##### IFM-2427 (DFV890) [CAS: 2271394-34-2]

IFM-2427 is an orally administered small molecule drug candidate that was developed by IFM Tre. Novartis acquired this molecule, and it was renamed DFV890. It is in phase 2 clinical trials for a number of NLRP3-associated diseases, such as symptomatic knee osteoarthritis (NCT04886258) and cardiovascular diseases (NCT06031844, NCT06097663). Phase 2 interventional clinical investigations for familial cold autoinflammatory syndrome (NCT04868968) and COVID-19 (NCT04382053) have been completed. The outcome of COVID-19 studies indicated that IFM-2427 has a protective effect against coronavirus-associated acute respiratory distress syndrome (CARDS). A phase 1-dose optimization study of this chemical for myelodysplastic syndrome (LR MDS) and lower-risk chronic myelomonocytic leukemia (LR CMML) is also underway (NCT05552469).

## Inferences and perspectives

Studies on the multifaceted nature of NLRP3 activation and its significance in the etiology of lifestyle diseases have revealed its pivotal role in immune responses and apoptosis, facilitated by the release of proinflammatory cytokines. NLRP3 is activated by an array of stimuli, including mitochondrial oxidation, ROS, and receptor expression. This review provides substantial evidence supporting the exacerbation of prevalent lifestyle diseases, such as obesity, diabetes, chronic respiratory diseases, oral diseases, and cardiovascular diseases, by the NLRP3 inflammasome. These findings indicate that the NLRP3 inflammasome plays a key role in driving pathophysiological changes at the cellular level in the context of the aforementioned diseases. This knowledge can pave the way for the development of innovative treatment strategies for a range of diseases. Further investigations are warranted to determine the involvement of other NLRs in lifestyle disorders. However, as multiple factors interfere with NLRP3 activation, such elucidation remains a challenge and needs to be further investigated for a “super-inhibition” approach of the NLRP3 inflammasome in all aspects.
